# Public perceptions of mortality studies in conflict-affected areas of the Eastern Mediterranean Region: an exploratory study

**DOI:** 10.1186/s13031-026-00798-x

**Published:** 2026-05-14

**Authors:** Esra Abdallah Abdalwahed Mahgoub, Ali Alshalah, Farah El Halabi, Manasik Mamoun, Azzam Ali Mohammad Fare’a, Leina Elomeiri, Sajjad Alazzawi, Abdirahman Khalif Mohamud, Awni Mokhtar Sallam Ali Awn, Mohammed Aref Ali Saeed Ahmed, Zahraa Zibara, Fadila Alhamwi, Noseiba Mahamoud Adam Abdelmoemen, Nour Jamal Houri, Mohamad Al Merhabi, Ola Ali Alftaeih, Mohamed Abdulkadir Ahmed Farah, Moneer Ali Abdallah Ali, Alessandra Ferrario

**Affiliations:** 1 Faculty of Medicine, Al Neelain University, Khartoum, Sudan; 2 One Percent Research Initiative, Khartoum, Sudan; 3 Communicable Diseases Control Center, Baghdad, Iraq; 4 UNICEF, Mbeya, Tanzania; 5 World Health Organization Regional Office of Africa, Brazzaville, Congo; 6 Resafa Directorate of Health, Baghdad, Iraq; 7 Africa Centres for Disease Control and Prevention, Addis Ababa, Ethiopia; 8 Jamhuriya Research Center, Jamhuriya University of Science and Technology, Mogadishu, Somalia; 9 Independent researcher, Sana’a, Yemen; 10 INTERSOS, Aden, Yemen; 11 Independent researcher, Beirut, Lebanon; 12 National Ribat University, Khartoum, Sudan; 13 Norwegian Refugee Council, Kampala, Uganda; 14 Sudan University of Science and Technology, Khartoum, Sudan; 15 Independent researcher, Neom, Saudi Arabia; 16 Personal Capacity, Bern, Switzerland

**Keywords:** Mortality, Conflict, Eastern Mediterranean Region, Public Perceptions, Data Collection

## Abstract

**Background:**

Mortality surveys in conflict-affected settings are essential for documenting the human cost of armed conflict, guiding the humanitarian response, and promoting accountability. The success of such studies relies on the participation of the affected populations. Yet, we know little about how the public in conflict-affected countries perceives such studies, their willingness to participate, or the barriers they face to participate. This study aimed to explore public perceptions, willingness and barriers to participate in mortality surveys in six conflict-affected countries of the Eastern Mediterranean Region.

**Methods:**

An exploratory, cross-sectional mixed-methods study was conducted in Sudan, Somalia, Syria, Lebanon, Iraq, and Yemen between March and June 2025. An online self-administered questionnaire captured both quantitative and qualitative data. Quantitative data were analyzed using descriptive statistics and bivariate tests, while qualitative responses underwent inductive content analysis.

**Results:**

Among 3455 participants (median age 29 years; 56.5% female), 94% (*n* = 3254) considered mortality surveys during armed conflicts important. Two-thirds (66.7%, *n* = 2306) expressed willingness to participate in mortality surveys, with a preference for electronic surveys. Willingness to participate was positively associated with older age, male sex, nationality, presence in the country during conflict, comfort with data sharing in social media, and absence of perceived cultural and religious barriers (*p* < 0.05). More than 40% of respondents reported trusting to provide death data to the government (44.3%, *n* = 1529) and international organizations (43.8%, *n* = 1515). Respondents reporting no trust to provide this data mentioned lack of credibility and political bias were the main reasons for distrust. Only 13.1% (*n* = 453) of the respondents perceived mortality surveys as harmful; key concerns included retaliation, political manipulation, and psychological distress. 19% (*n* = 658) cited cultural or religious barriers, most linked to sectarianism, honor, and the sanctity of death, as an obstacle to participation.

**Conclusion:**

Perceptions captured across the six conflict-affected EMR countries among the digitally-active adult respondents reflect both recognition of the importance of mortality surveys and concerns about safety, trust, and cultural sensitivities. Enhancing confidentiality, engaging trusted institutions in data collection, and adopting culturally sensitive approaches are critical to strengthening participation and ensuring the ethical implementation of mortality surveys in conflict zones.

**Supplementary Information:**

The online version contains supplementary material available at 10.1186/s13031-026-00798-x.

## Introduction

Over the past two decades, the WHO Eastern Mediterranean Region (EMR)^1^ has evolved into one of the most complex and protracted humanitarian settings globally. A combination of armed conflicts, weak governance, economic instability, and climate-related shocks has produced overlapping crises profoundly affecting public health and human development. According to the World Health Organization (WHO), thirteen of the region’s 22 countries are directly or indirectly affected by conflict, while the World Bank classifies nine as fragile or conflict-affected states [1, 2]. Several EMR countries have faced repeated disruptions to essential services, including healthcare, education, and infrastructure [1, 3–5]. These interlocking crises have contributed to what scholars increasingly describe as “syndemic”, where violence, infectious diseases, food insecurity, and psychological trauma interact to magnify one another’s effects [1, 6].

The consequences of these crises to human life loss are both immediate in the form of direct fatalities from violence and long-term as indirect deaths resulting from malnutrition, preventable diseases, and the collapse of public health systems [4, 7] in conflict-affected settings. For instance, in Yemen, the war had resulted in an estimated 377,000 deaths by the end of 2021, more than half of which were caused by conditions such as hunger, cholera, and disrupted maternal care [8]. Understanding the scale and nature of these losses requires reliable mortality data to inform international humanitarian and human rights responses [9–11]. Civil registration and vital statistics systems record births and deaths and are the foundation for mortality estimation. However, in most conflict-affected countries, civil registration systems often deteriorate as conflict progresses [12] and may have been already weak before the conflict started. As a result, retrospective mortality surveys, typically conducted through household interviews, remain the primary method for estimating death tolls, including in countries of the EMR [15, 16].

Despite their importance, retrospective mortality surveys face significant methodological and ethical challenges related to participation and disclosure when asking respondents about deaths within the household. While the literature suggests reasons for non-participation, limited evidence is available on the willingness to participate in such surveys. Commonly discussed reasons for refusal to participate or hide information include fear of retaliation [13] and concerns that their reputation could be endangered [14]. This reluctance is likely to be compounded by a lack of trust in institutions, often rooted in cultural or religious beliefs [15, 16]. Furthermore, individuals may intentionally conceal specific types of deaths, such as those of combatants [14, 17, 18] or fatalities related to torture, detention, and sexual violence [14]. These concerns can even manifest as hostile or threatening behavior towards data collectors [12, 18]. Despite a growing body of research on mortality surveys in the EMR, little is known about the public’s perspectives towards studies trying to estimate conflict-related deaths, willingness to share death-related information or the conditions under which they feel comfortable doing so. This study addresses this gap by examining attitudes toward sharing conflict-related mortality surveys, identifying barriers to participation, and assessing which institutions individuals trust with such sensitive information. By exploring these factors, we aim to identify ways to enhance public participation in mortality surveys, ultimately leading to better-informed policy and humanitarian action.

## Materials and methods

All the methods in this study adhered to the Checklist for Reporting Results of Internet E-Surveys (CHERRIES) guideline (Additional file 1). Specifically, the study addressed CHERRIES-recommended elements related to survey design, development and pre-testing, survey type, recruitment and dissemination, survey context, data collection timeframe, questionnaire structure, response review functionality, and ethical considerations including approval, informed consent, voluntariness, and incentives.

### Study design and population

We conducted a cross-sectional, mixed-methods study in six countries of the EMR with a history of armed conflict/ protracted conflicts during the past 15 years, as defined in the Technical Guidance Note on SDG Indicator 16.1.2 [19]. The six countries are Sudan, Somalia, Syria, Lebanon, Iraq, and Yemen. The initial list also included the occupied Palestinian territories and Libya; however, due to the limited number of responses received (57 from Libya and 30 from Palestine), we did not include them in the analysis.

An online survey was administered to the general population using a social media platform. To be eligible to participate in the study, respondents had to be at least 18 years of age, from any of the included countries, regardless of current residence, and users of any of the social media platforms used to disseminate the survey (Facebook, WhatsApp, and Telegram).

## Sampling

The sample size for each country was calculated using Cochran’s formula, assuming a 95% confidence level, a 50% expected outcome, and a non-response rate of 40%. The required sample size was 640 per country; therefore, a total of 3840 participants across the six countries. There were fewer than 640 respondents in Syria (*n* = 417) and Somalia (*n* = 349); however, given the exploratory nature of the study, they are included in the analysis. The final number of respondents was 3455.

Convenience sampling was employed to select participants via social media, complemented by referral sampling (participants were asked to share the questionnaire with their networks).

## Data collection

We conducted an open survey using an online, self-administered questionnaire. The questionnaire was initially developed in Arabic. Questions were informed by a literature review, the research team’s previous work [20] and the authors’ own knowledge of the region’s culture and norms. The ten authors then reviewed the questionnaire and provided feedback on wording and clarity, cultural sensitivity, selected constructs, and item placement. For Somalia and Lebanon, we translated the Arabic questionnaire to Somali and English languages to be used in the two respective countries. Translation and back translation of the questionnaire were conducted by bilingual researchers.

We piloted the questionnaire on 80 respondents (10 from each country), from different genders, age groups, and education levels. The questions were then refined based on the pilot survey respondents’ feedback. The questionnaire is available in Additional file 2 and includes a total of 23 questions, five open-ended (qualitative) questions, and 18 closed-ended (quantitative) items divided over three sections. The demographic section recorded information on the respondent’s age, sex, nationality, education level, and presence in the country during the period of war or armed conflict. The second section focused on the participants’ attitudes towards sharing mortality data, and the third section covered the participants’ perceived barriers to sharing mortality data. The questionnaire was administered as a one-page Google Form.

All questions (except the open-ended questions) were mandatory to ensure complete responses. Participants could review and edit their answers before submitting. A total of 35 local data collectors (18 members of the research team and 17 volunteers included in the acknowledgements) disseminated the questionnaire via Facebook, WhatsApp, and Telegram in their respective countries. To maximize variation among participants, we used diverse geographic and thematic targets to include people from different geographic areas, genders, age groups, shared interests, and professions. The team encouraged respondents to share the questionnaire with their circles to disseminate the survey further. Data collection took place from March 17, 2025, to June 10, 2025.

### Data analysis

We exported the responses from Google Forms to a Microsoft Excel^®^ workbook. Responses in Somali language were translated to English by two Somali native speakers, part of the research team. Multiple-choice questions were converted to binary variables (1 = True, 0 = False). Quantitative data were initially cleaned in MS Excel and later wrangled and analyzed in R (version 4.4.1). We used descriptive statistics, including frequencies and percentages. For bivariate analysis, we used the Chi-squared and Mann-Whitney tests to assess associations between sociodemographic and attitude variables. The significance level was set at alpha = 0.05.

For the qualitative data, we conducted a content analysis of Arabic and English responses (including translated Somali responses) in MS Excel. We developed a codebook where categories were developed inductively. This allowed us to capture participants’ perspectives without relying on predefined classifications, and to complement and interpret the quantitative findings. Three researchers conducted the qualitative analysis. First, all three independently reviewed the responses to each open-ended question and proposed initial codes. These were then discussed collectively and refined into a common codebook. Next, the five questions were divided among the researchers so that each answer was reviewed by two researchers. To enhance reliability, the coded responses were subsequently exchanged so that each response was reviewed by a different researcher than the original coder, and discrepancies were identified. These discrepancies were resolved through group discussion. Finally, one researcher conducted a comprehensive review of the entire coded dataset to ensure consistency across categories. The results were presented as categories with frequency counts to highlight their relative prominence. This approach enabled us to both capture the breadth of perspectives and quantify their distribution.

## Results

### Sociodemographic characteristics of participants

There were 3455 respondents from six countries. Specifically, 681 responses came from Lebanon, 675 from Sudan, 670 from Yemen, and 663 from Iraq. We did not reach the desired sample size in Syria and Somalia, which had 417 and 349 responses, respectively. The median age of participants was 29 years (IQR 23–37), with ages ranging from 18 to 85 years. More than half of the respondents were female (56.5%, *n* = 1953), and 83% (*n* = 2868) were in their countries during the period of armed conflict. More than half of the respondents held a bachelor’s degree (58.5%, *n* = 2021), while only 454 (13.2%) had a secondary school education or lower. The sociodemographic information of participants is presented in Table 1.

**Table 1:** Sociodemographic information of participants (*N* = 3455)

	Frequency	Percentage
Age Median (IQR) years*	29 (23–37)	
**Gender**		
Female	1953	56.5%
Male	1502	43.5%
**Nationality**		
Lebanese	681	19.7%
Sudanese	675	19.5%
Yemeni	670	19.4%
Iraqi	663	19.2%
Syrian	417	12.1%
Somali	349	10.1%
**Education**		
Never attended school	19	0.6%
Primary	51	1.5%
Secondary	384	11.1%
Diploma	257	7.4%
Bachelor’s	2021	58.5%
Master	525	15.2%
Doctorate or higher	198	5.7%
**Present in the country during the conflict**		
Yes	2868	83.0%
No	587	16.99%

*N = 3411

## Perceived importance of mortality surveys

When asked about the importance of mortality surveys during conflict, most participants (94.2%, *n* = 3254) considered it very important or important. The majority (84.6%, *n* = 2923) thought that estimating the number of deaths during wars or armed conflicts could help improve humanitarian efforts in affected areas. 2543 (74%) believed that collecting mortality data could help achieve legal justice and hold those responsible accountable. 2026 (58.6%) believed that collecting such information could help prevent future deaths (Table 2). There was a statistically significant difference between the countries in the perceived benefit of mortality surveys in preventing future deaths and achieving justice (*p* < 0.001) (Additional file 3, Figs. 1 and 2).

**Table 2:** Perception about the importance of mortality surveys among the participants (*N* = 3455)

Question	Rnsesespo		
	Very important/ Important *n* (%)	Neutral *n* (%)	Not important/Not important at all *n* (%)
To what extent do you think mortality surveys in war or conflict zones is important?	3254 (94.2)	135 (3.9)	66 (1.9)
	**Yes** **n (%)**	**Not sure** **n (%)**	**No** **n (%)**
Do you believe that mortality surveys during wars or armed conflicts can help improve humanitarian efforts?	2923 (84.6)	418 (12.1)	114 (3.3)
Do you believe that mortality surveys during wars or armed conflicts can help achieve legal justice and hold perpetrators accountable?	2543 (73.6)	581 (16.8)	331 (9.6)
Do you believe that mortality surveys during wars or armed conflicts can help prevent future deaths?	2026 (58.6)	848 (24.5)	581 (16.8)

**Fig. 1:**
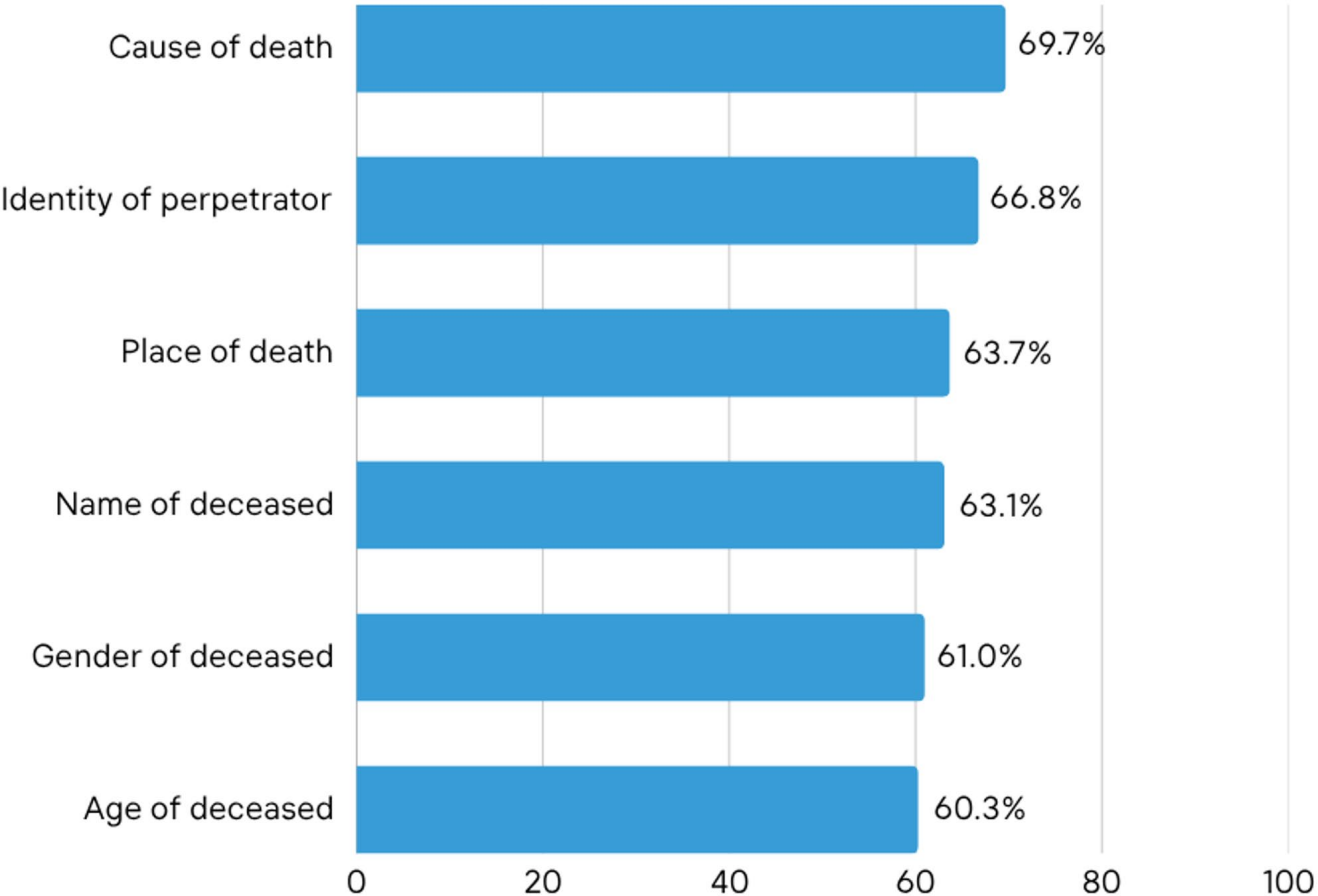
Types of mortality data participants are willing to share (*N* = 2306)

**Fig. 2:**
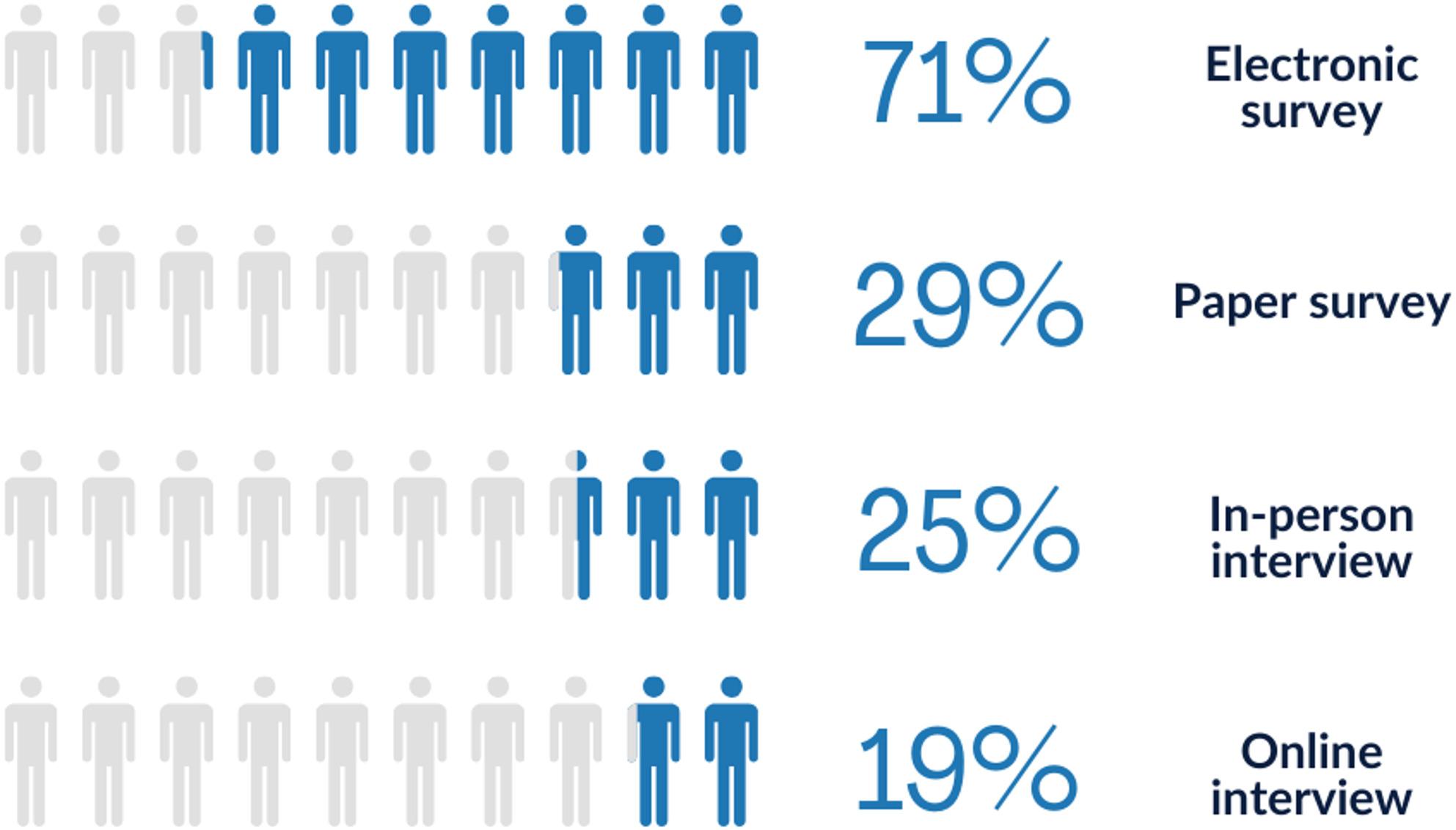
Preferred ways to share mortality data (*N* = 2306)

2064 participants elaborated on the reasons they considered collecting such data important (optional question). The majority (79.1%, *n* = 1633) believed that collecting mortality data is important for monitoring and documenting the effects of conflict, including human and asset losses. Additionally, 317 (15.7%) thought this data are important to support affected families by providing compensation and preserving their right to know the fate of missing persons, while 255 (12.4%) considered it important to ensure legal accountability and strengthen human rights. None of the respondents who reported that data were not important/not important at all provided reasons behind their perceptions. Further details on the qualitative themes are available in Additional file 3, Tables 4 and 5.

## Willingness to share mortality data

Two-thirds of the study respondents (66.7%, *n* = 2306) were willing to participate in a mortality survey during war in their country, 708 (20.5%) were unsure, and 441 (12.8%) were unwilling. Most of those who were not willing to participate (86.4%, *n* = 381) noted that collecting data about deaths during wars was important or very important. Similarly, most respondents who were unsure about participating (92.1%, *n* = 652,) reported that it was important or very important to do so. Among those willing to participate (*n* = 2306), more than 60% of respondents were willing to share additional data elements surrounding the reported deaths, the most frequent being cause of death (69.7%, *n* = 1607) and the identity of the perpetrator (66.8%, *n* = 1540) (Fig. 1). Most participants preferred to share data via electronic surveys (71.3%, *n* = 1644), while less than one in five chose online interviews (18.9%, *n* = 436) as the preferred method (Fig. 2).

Overall, 40% (*n* = 1381) of participants felt either very comfortable or somewhat comfortable sharing mortality data during the war or armed conflict through social media platforms, compared to 26.4% (*n* = 913) who reported feeling uncomfortable or somewhat uncomfortable, and 33.6% (*n* = 1161) who remained neutral. Willingness to participate in a mortality survey during war or armed conflict was significantly associated with participants’ age, sex, nationality, presence in the country during the period of war or armed conflict, their perception of sharing mortality data on social media platforms, and perceived cultural or religious beliefs that may affect the collection of mortality data in their country.

Respondents who were willing to participate in such surveys were older than those who were not willing or unsure (*p* < 0.001). Males were more willing to participate compared to females (*p* = 0.008). Additionally, nationality was strongly associated with willingness to participate (*p* < 0.001). Sudanese participants showed the highest willingness to participate (77.5%), followed by Iraqis (73.6%), Syrians (71.7%), and Yemenis (69.7%). In contrast, Somali (55.3%) and Lebanese (49.3%) respondents reported comparatively lower levels of willingness but still more or close to 50%. A greater willingness to participate was also seen among those who were in-country during the war or armed conflict period (*p* = 0.003), those comfortable with sharing mortality data on social media (*p* < 0.001), and those who did not believe mortality data collection was harmful and perceived no cultural or religious barriers (*p* < 0.001) (Additional file 3, Table 1).

### Participants’ perception of local community leaders and the organization they trust

Overall, 43.3% (*n* = 1497) of respondents believed it was important to obtain permission from local community leaders before conducting mortality surveys in their community (Table 3). This belief was stronger among males than females (*p* = 0.015) and participants who believed that cultural or religious beliefs make mortality data collection difficult (*p* < 0.001). Nationality was strongly associated with this perception (*p* < 0.001), with the highest frequency among Somali (56.4%) and Yemeni (55.2%) participants, while Iraqi participants showed the lowest percentage (29.1%) (Additional file 3, Table 2).

Most respondents trusted governmental organizations (44.3%, *n* = 1529) and international organizations (43.9%, *n* = 1515) to share death data with during war or armed conflict, followed by local academic researchers (30.8%, *n* = 1063), international academic researchers (21.7%, *n* = 749), local NGOs (19.3%, *n* = 666), and community leaders (16.1%, *n* = 555) (Table 3).

**Table 3:** The attitudes toward community permission and trust in organizations collecting mortality data

	Frequency	Percent
**Is it important to obtain permission from local community leaders before mortality surveys?**		
Yes	1497	43.3
Not sure	768	22.2
No	1190	34.4
Entities that participants trust to share mortality data with during war or armed conflict *		
Governmental organizations	1529	44.3
International organizations	1515	43.8
Local academic researchers	1063	30.8
International academic researchers	749	21.7
Local non-governmental organizations	666	19.3
Local community leaders	555	16.1
I do not trust any of them	534	15.5

*Percentages may exceed 100% as participants could select more than one option

1828 participants explained why they trusted these organizations (optional qualitative question). The most frequently mentioned reasons were impartiality, credibility, trustworthiness, transparency, and the institutions’ good reputation (46.3%, *n* = 846). The second most common reason was that the selected entity possessed governmental, legal, or official authority and represented the people (12.9%, *n* = 237). Additionally, 172 participants (9.4%) stated that the entities they chose as trusted had the power to enact change and to disseminate information (Additional file 3, Tables 4 and 5).

**Fig. 3:**
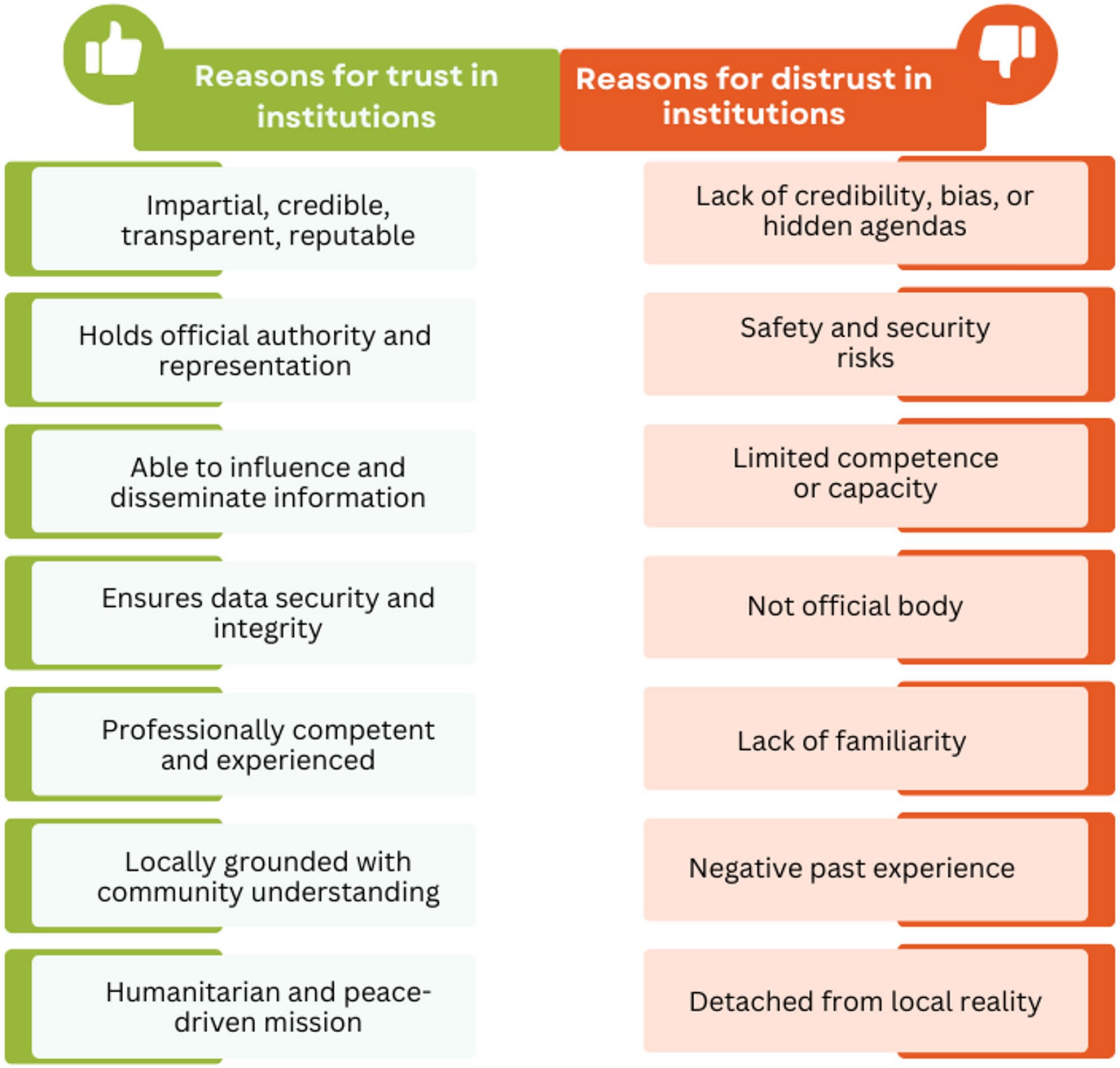
The reasons for trust or distrust entities to share mortality data with during war or armed conflict

1746 participants also explained why they did not trust certain organizations (optional qualitative question). 1130 responses (64.7%) cited a lack of credibility, ethical misconduct, or political bias or a hidden agenda as reasons for distrust. 129 (7.4%) respondents also mentioned security and safety concerns for the informants and their close ones as reasons for distrusting certain organizations or actors (Fig. 3) (Additional file 3, Tables 4 and 5).

Younger age (*p* < 0.001) and men (*p* = 0.002) were more likely to be willing to share mortality data during conflict with governmental institutions. Trust also varied significantly by nationality (*p* < 0.001) (Additional file 3, Table 3).

### Perspectives on the potential harm and barriers of mortality surveys

A minority of respondents reported that collecting mortality data could be harmful (13.1%, *n* = 453), while 56.7% (*n* = 1960) thought it was not, and 30.2% (*n* = 1042) were not sure. Of those who believed collecting mortality data during conflict was harmful, 221 responded to the optional question specifying the type of harm. Retaliation and political manipulation were the most commonly perceived harms, reported by 121 (54.8%) and 78 (35.3%) participants, respectively. Moreover, 45 (20.4%) mentioned social and psychological harm to families, as the recollection of their relatives’ deaths could be distressing for some (Additional file 3, Table 3).

There was a statistically significant difference between countries in the perceived harm of collecting mortality data in conflict-affected areas (*p* < 0.001), with participants from Somalia reporting most frequently perceived harm (23.5%) (Additional file 3 Fig. 3**).**

**Fig. 4:**
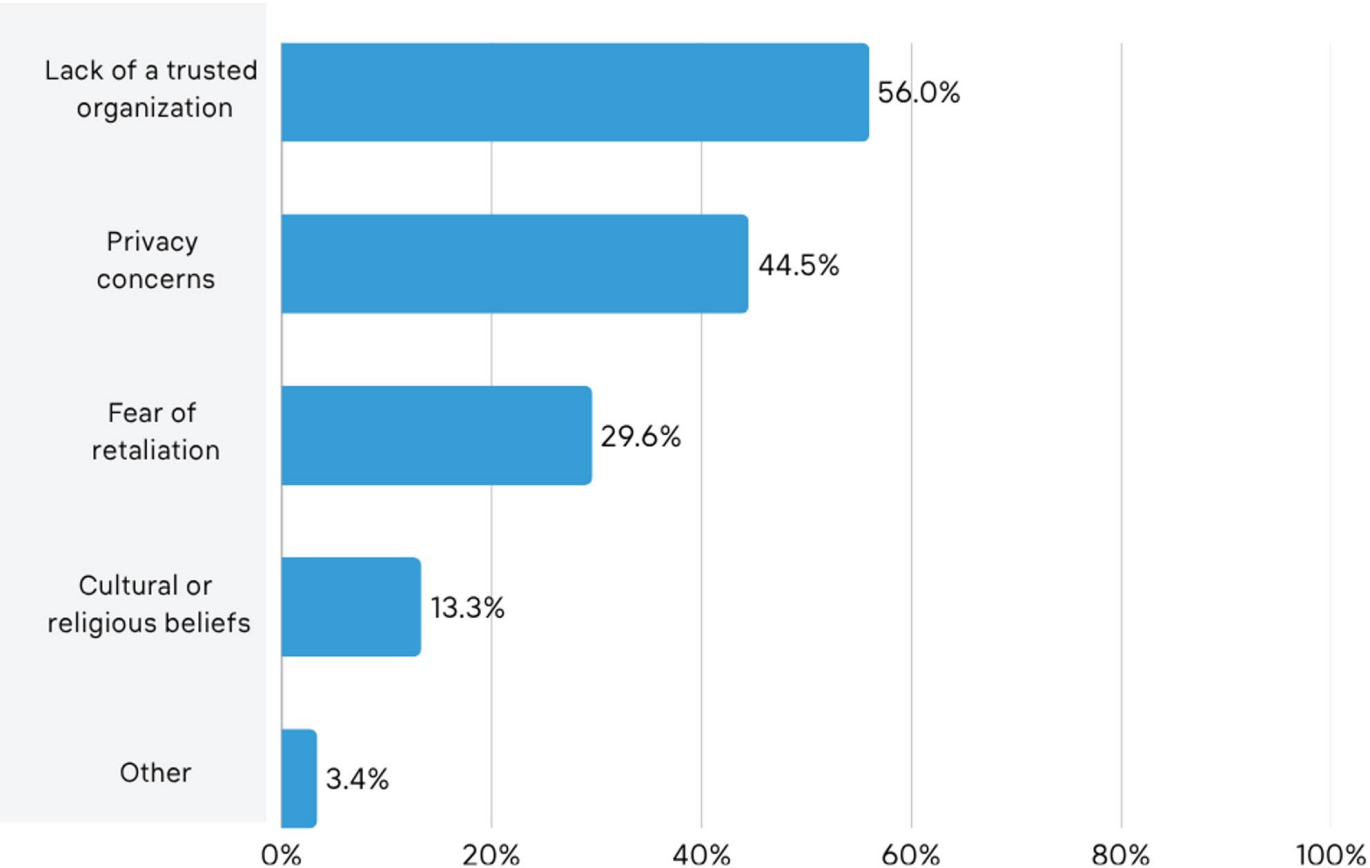
The barriers that prevent people from sharing information about deaths during conflict

Lack of trust in the organizations collecting the data (56.0%, *n* = 1936) and privacy concerns (44.5%, *n* = 1536) were the most common reasons for reluctance to share mortality data during conflict (Fig. 4).

A minority of participants (19%, *n* = 658) stated that cultural or religious beliefs made collecting such data difficult in their country (Additional file 3, Fig. 4**).** The prevalence of this belief was highest within the Somali community, where nearly a quarter (23.2%) acknowledged these obstacles, followed by Iraqi and Yemeni participants, while only 11.9% of Sudanese participants identified such barriers.

Among respondents mentioning cultural and religious beliefs as barriers to data collection, 188 provided further details by answering the optional open-ended question. 121 (64.4%) participants mentioned not wanting to aggravate the existing divisions within groups (stemming from ethnic, tribal, sectarian, and religious diversity). Cultural secrecy and fear of defamation were mentioned by 30 participants (15.9%) and included social stigma, shame, and defamation associated with naming the deceased or revealing the causes of death. This category reflected deeply held values around family and tribal honor, respect for privacy, and the protection of the deceased’s and their relatives’ reputations. Privacy of death and the sanctity of the deceased were mentioned by 19 participants (10.1%). This category reflects the belief that discussing the cause of death or conducting post-mortem procedures is a violation of religious norms and an intrusion on the personal and familial dignity of the deceased (Additional file 3, Tables 4 and 5).

## Discussion

This cross-sectional study investigated digitally connected adults’ perceptions of mortality surveys in six conflict-affected countries in the Eastern Mediterranean Region. Most respondents supported collecting mortality data during war or armed conflict. Two-thirds of the participants were willing to take part in a survey, preferably an online survey. The study also showed that governmental and international organizations were the most trusted entities with which participants were willing to share mortality data. Lack of trust in the entity collecting data and privacy concerns were the main reasons for not wanting to share information about death in conflicts.

Two-thirds of the participants in our study were willing to participate in mortality surveys. While this percentage is encouraging and reflects community openness, this willingness may not translate into actual participation, especially during active conflict and if detailed information about the person who died is asked (e.g. name, age, gender, place of death). The fact that 86% of those unwilling to participate still valued the importance of collecting this information may indicate that barriers to participation outweighed the perceived importance of documenting deaths during conflict. Our study showed a significantly higher willingness to participate in mortality surveys among individuals who were present in the country during the war or armed conflict. This suggests that direct exposure to the conflict may have fostered a sense of personal responsibility to document the horrific effects of the war on their community [21]. Additionally, there was a significant difference in willingness to participate across countries. This may be linked to varying perceptions of the importance of documenting deaths, possible harm, and cultural barriers.

The data reveal a critical distinction between the perceived immediate humanitarian and future strategic values of the information. While a large portion of respondents (84.6%) saw the data as important to improve humanitarian efforts, only 58.6% considered it useful to prevent future deaths. Furthermore, only 108 participants viewed the data as essential for peacebuilding and political action. In contrast, a larger group of 317 participants focused on the more immediate, tangible impact, valuing its ability to support affected families through compensation and preserving their right to know the fate of missing persons.

Our study showed that willingness to participate in mortality surveys does not necessarily mean that respondents are willing to share all the information around deaths. Willingness to share specific elements (e.g. cause of death, age of deceased, gender) ranged from 60% to 70%. Detailed information about each death is crucial for reporting SDG Indicator 16.1.2, as it helps prevent double-counting and ensure consistency across data from various sources [19]. Still, this needs to be weighed against the expected response rate if the conflict is active. Our findings show that participants were less willing to disclose a person’s age and gender than the identity of the perpetrator. There are various possible explanations. For example, deaths of young males may be concealed, as they are often perceived as active participants in conflict, and acknowledging their deaths might bring stigma or suspicion upon the family [14, 17, 22]. Reporting the death of a female can also be sensitive, as it may be linked to issues of family honour or fears of sexual violence [14, 22]. Likewise, the death of a child is an emotionally painful subject, and families may avoid disclosing it to protect themselves from grief or due to cultural beliefs surrounding childhood mortality [18, 23].

44% of participants reported trusting governmental and international organizations to share conflict-related mortality data. This aligns with the stated importance of credibility and reputation as a source of trust, in addition to the power to change things, which might be easier for institutions to establish than for individual researchers. Participants may also question the agenda of researchers and the usefulness of their data. While local academic institutions are best positioned to lead mortality estimation studies due to their expected impartiality and knowledge of country and language [24], our findings point to the need to build greater public trust. Partnerships with governmental and international organizations may ameliorate this on the condition that they do not affect impartiality. Interestingly, Lebanese participants in our study demonstrated the highest level of trust in sharing information with governmental institutions. This finding is particularly notable as it appears to contradict a regional trend: Arab Barometer Wave 7 data show that Lebanon typically has the lowest levels of government trust across the studied countries in the region [25]. Our results may thus reflect this preference as the most viable option for participants compared to other available channels. Participants from Yemen showed the lowest level of trust in government. This could be linked with the fragmentation of state institutions and the challenges the government faces in implementing policies and maintaining effective governance [26].

Less than half of the participants (43.3%) thought it was important to obtain permission from local community leaders before collecting information on deaths in their community. This was most frequently mentioned in Somalia and Yemen and reflects governance arrangements. In Somalia, the clan system is the central organizing principle of society and politics [28], and in Yemen, tribal leaders often hold considerable power [26].

Only a minority of participants (13.1%) regarded the collection of conflict mortality data as harmful. The types of harm they identified highlight substantial risks for researchers to consider. The most common fear was retaliation. Another reported harm was the manipulation of data by interested parties, which has been reported before [27, 28]. Some participants also felt that such data could pose a national security threat by escalating tensions between warring factions and leading to further cycles of revenge. These perceptions echo the most frequently cited barriers to participation in mortality surveys identified in our study: lack of trust in the entity collecting the data and privacy concerns. This places a great responsibility on researchers to ensure participants’ safety. Best practices require maximizing confidentiality [29], respecting privacy and anonymity [30], and avoiding questions about sensitive topics that could put respondents at risk [31]. This can be achieved by tailoring measures according to the security level in place. For example, encrypted online survey tools can be utilized to minimize participant contact in active conflict settings. For on-ground data collection, digital applications may be preferable to paper-based methods because they enable immediate anonymization, secure server uploads, and deletion of local copies to mitigate unauthorized access in the event of device confiscation. Moreover, using neutral interview locations and local collectors may minimize the survey’s visibility and reduce social risks for respondents. In some cases, it is advisable to exclude detailed questions about names, perpetrators, or specific locations to prioritize participants’ safety. A third type of harm mentioned was the likely psychological distress from asking families to recall traumatic events. For some traumatized individuals, silence may be a coping mechanism rather than a mere survival strategy [30]. Therefore, researchers must exercise restraint and sensible judgment [30]. Efforts should be made to identify and, if possible, exclude particularly vulnerable individuals from the research [29]. Researchers should also consider providing psychological support or referrals to those in need. All these measures call for training of data collectors, and adoption of best research practices in conflict settings [29].

Only 19% of participants perceived cultural and religious beliefs as a barrier to mortality surveys. These could be particularly significant for a specific subset of the population. Cultural and religious issues around collection of mortality data are not unique to our study. Similar factors have been noted in Sudan [23, 32, 33] and Iraq [15]. The most frequently mentioned cultural factors were ethnic, tribal, sectarian, and religious factors. As a result, communities may withhold information to protect their own members, avoid blame, or prevent the perceived victimization of their social group. Another significant factor mentioned was fear of defamation and preservation of honor. This reflects deeply held values around family and tribal honor, respect for privacy, and the protection of the deceased and their relatives’ reputations. Additionally, deeply held beliefs about the sacredness of death may lead families to consider it a private matter that should not be used for public documentation. For some, a belief in fatalism might cast documentation as pointless or disrespectful. Sensitivity regarding female deaths, including those related to sexual assaults and honor crimes, was also mentioned. This is consistent with findings from previous studies in Iraq [22] and Syria [14]. These cultural nuances underscore that researchers must demonstrate a high degree of cultural humility and respect beyond transactional approaches to data collection. A more in-depth exploration of these factors would be beneficial to inform the development of more culturally sensitive and effective methodologies for humanitarian and health-related research in conflict zones.

### Limitations

This study has several important limitations that should be considered cautiously when interpreting the findings. The nonprobability sampling strategy limits the extent to which the findings can be generalized. The use of social media as the primary data collection channel in this study led to a sample that disproportionately included younger and more educated individuals. As such, their estimates of willingness to participate in mortality surveys, trust in institutions, and their perception of harm or barriers are likely influenced by this selection bias and may not accurately represent the broader population. The use of an online tool may limit participation among individuals with lower literacy levels, limited internet access, and, in some cases, older adults and those living in rural areas. While these groups are not necessarily excluded, they may be underrepresented. This is particularly important, as such populations may experience heightened vulnerability during conflicts and could hold perspectives that differ from those captured in the sample. However, the use of the online tool was a pragmatic way to reach respondents, some of whom live in countries with active conflict, and to cover different areas within a country. Another aspect pertinent to reliance on social media for data collection is the introduction of self-selection and self- desirability bias. Individuals who chose to participate may already be active on social media and engaging in humanitarian, academic, or civil discussion, resulting in normative or socially acceptable attitudes that highlight justice or humanitarian benefit. Future research is needed to study other factors that might affect willingness to participate in mortality data collection but were not included in the study such as ethnicity, political alignment, sectarian affiliation, nature and duration of conflict in each country, and political freedoms. All may affect participants’ decisions to respond to a survey. Despite these limitations, the study offers unique and valuable insights into the critical factors to consider when conducting mortality surveys in the studied countries and similar environments.

## Conclusion

This study shows that digitally-active adult respondents in studied conflict-affected countries of the Eastern Mediterranean Region generally recognize the importance of collecting mortality data and are willing to participate in research. The preference for electronic surveys indicates openness to digital approaches. With varying trust in different institutions, more research is needed to understand who are the best entities and arrangements to ensure high response rates.

At the same time, the findings highlight concerns affecting respondents’ willingness to participate in mortality surveys. These include fear of retaliation and data manipulation, psychological distress, as well as cultural and religious sensitivities surrounding death. These concerns underscore the need for researchers to adopt approaches that safeguard confidentiality, respect cultural norms, and ensure impartiality.

Mortality surveys remain essential for documenting the human cost of war, informing humanitarian and recovery responses, and supporting accountability efforts. The findings of this study can help inform future study design and data collector training to mitigate some of the barriers identified.

## Supplementary Information

Below is the link to the electronic supplementary material.

Supplementary Material 1.

Supplementary Material 2.

Supplementary Material 3.

## Data Availability

The datasets analyzed during the current study are available from the corresponding author upon reasonable request.

## Footnotes

1 The region comprises 22 countries and territories: Afghanistan, Bahrain, Djibouti, Egypt, Jordan, Kuwait, Iran (Islamic Republic of), Iraq, Lebanon, Libya, Morocco, the occupied Palestinian territory, Oman, Pakistan, Qatar, Saudi Arabia, Somalia, Sudan, Syrian Arab Republic, Tunisia, Yemen, and United Arab Emirates.

## Abbreviations

CHERRIES: Checklist for Reporting Results of Internet E-Surveys
EMR: Eastern Mediterranean Region
WHO: World Health Organization
