# Supplementary material for: Public perceptions of mortality studies in conflict-affected areas of the Eastern Mediterranean Region: an exploratory study
Source: Confl Health. 2026 May 14;20:62. doi: 10.1186/s13031-026-00798-x (PMC13352670; doi:10.1186/s13031-026-00798-x)
Supplement: Supplementary file 2 — Supplementary Material 2. [file 13031_2026_798_MOESM2_ESM.docx]

**Questionnaire about Public Perceptions of Mortality Studies in Conflict-Affected Areas of the Eastern Mediterranean Region**

**Section 1: Demographic Information**

1. What is your age?
2. What is your gender?
   - ☐ Male
   - ☐ Female
3. Please specify your nationality:
   - ☐ Sudanese
   - ☐ Somali
   - ☐ Syrian
   - ☐ Lebanese
   - ☐ Palestinian
   - ☐ Iraqi
   - ☐ Libyan
   - ☐ Yemeni
4. What is the highest level of education you have attained?
   - ☐ No formal education
   - ☐ Primary school
   - ☐ Secondary school
   - ☐ Bachelor's degree
   - ☐ Master's degree
   - ☐ Doctorate or higher
5. Have you been present in your country during the period of war or armed conflict (even for a short duration)?
   - ☐ Yes
   - ☐ No

**Section 2: Attitudes Toward Sharing Mortality Data**

1. To what extent do you think collecting mortality data in war or conflict zones is important?
   - ☐ Very important
   - ☐ Important
   - ☐ Neutral
   - ☐ Not important
   - ☐ Not important at all
2. Why do you think collecting mortality data during wars and armed conflicts is important?
   - **[Open-ended response]**
3. Do you believe that studies estimating death tolls during wars or armed conflicts can help improve humanitarian efforts?
   - ☐ Yes
   - ☐ No
   - ☐ Not sure
4. Would you be willing to participate in a survey about war-related mortality in your country?
   - ☐ Yes
   - ☐ No
   - ☐ Not sure
5. If you answered "Yes" (to question 9), what types of data would you be willing to share regarding deaths in your community? (Select all that apply)

- ☐ Name of the deceased
- ☐ Age of the deceased
- ☐ Gender of the deceased
- ☐ Location of death
- ☐ Cause of death
- ☐ Number of deaths
- ☐ Perpetrator of the death

1. If you answered "Yes" (to question 9), what method do you prefer for sharing this data?

- ☐ Face-to-face interview
- ☐ Online interview
- ☐ Paper-based survey
- ☐ Electronic survey

1. How comfortable are you with sharing mortality data during wars or armed conflicts on social media platforms (such as Facebook, WhatsApp, Twitter)?

- ☐ Very comfortable
- ☐ Somewhat comfortable
- ☐ Neutral
- ☐ Somewhat uncomfortable
- ☐ Very uncomfortable

1. Which of the following entities would you trust to share mortality data with during war or armed conflict?

- ☐ Government entities
- ☐ International organizations (e.g., United Nations, World Health Organization)
- ☐ Non-governmental organizations (NGOs)
- ☐ Local community leaders
- ☐ Local academic researchers
- ☐ International academic researchers
- ☐ I do not trust any of them
- ☐ Other: ___________

1. Why do you trust the entity you selected?

- **[Open-ended response]**

1. Why do you not trust the other entities?

- **[Open-ended response]**

1. Do you think it is important to obtain permission from local community leaders (tribal chiefs, imams, religious figures, or other notable figures) before collecting mortality data in their community?

- ☐ Yes
- ☐ No
- ☐ Not sure

1. Do you believe that collecting mortality data during wars or armed conflicts can help prevent future deaths?

- ☐ Yes
- ☐ No
- ☐ Not sure

1. Do you believe that collecting mortality data during wars or armed conflicts can help achieve legal justice and hold perpetrators accountable?

- ☐ Yes
- ☐ No
- ☐ Not sure

1. Do you think collecting this type of data could be harmful in any way?

- ☐ Yes
- ☐ No
- ☐ Not sure

1. If you answered "Yes," how could it be harmful?

- **[Open-ended response]**

**Section 3: Barriers to Sharing Mortality Data**

1. What barriers might prevent you from sharing mortality data during a conflict? (Select all that apply)

- ☐ Lack of trust in data-collecting entities
- ☐ Privacy concerns
- ☐ Fear of retaliation
- ☐ Cultural or religious beliefs
- ☐ Other: ___________

1. Do you think there are cultural or religious beliefs that make it difficult to collect mortality data in your country?

- ☐ Yes
- ☐ No
- ☐ Not sure

1. If you answered "Yes," can you specify these beliefs?

- **[Open-ended response]**
