# Supplementary material for: Public perceptions of mortality studies in conflict-affected areas of the Eastern Mediterranean Region: an exploratory study
Source: Confl Health. 2026 May 14;20:62. doi: 10.1186/s13031-026-00798-x (PMC13352670; doi:10.1186/s13031-026-00798-x)
Supplement: Supplementary file 3 — Supplementary Material 3. [file 13031_2026_798_MOESM3_ESM.docx]

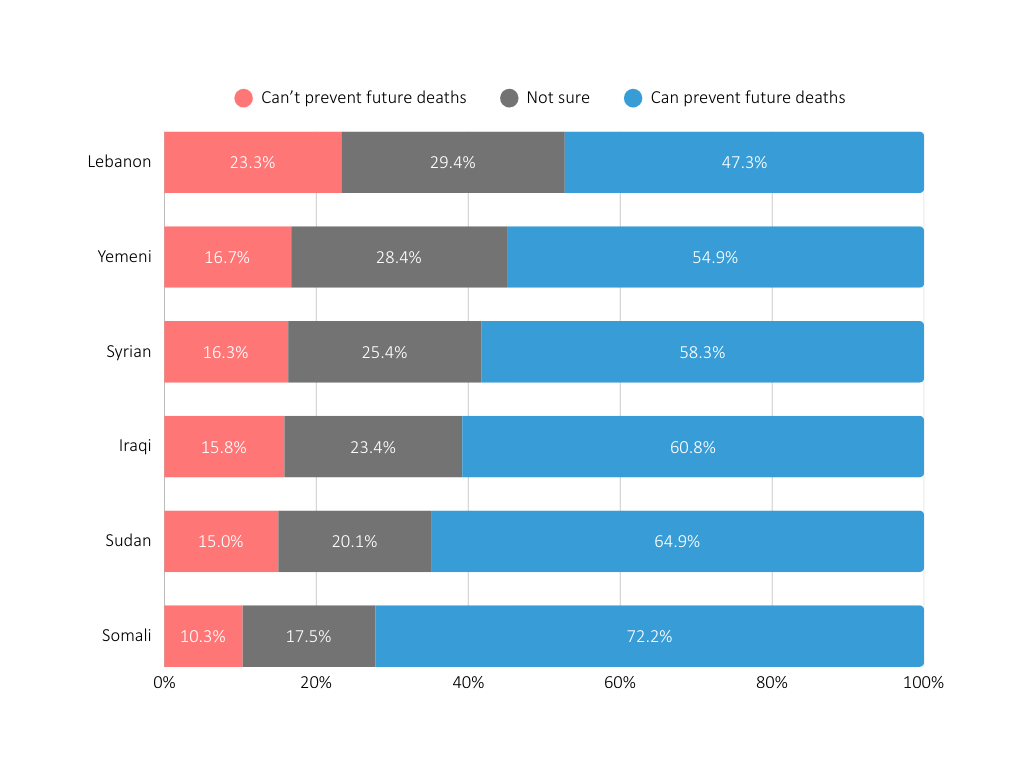
**Figure 1. Perception that collecting mortality data in conflict-affected areas can help prevent future deaths by country (p < .001)**

**
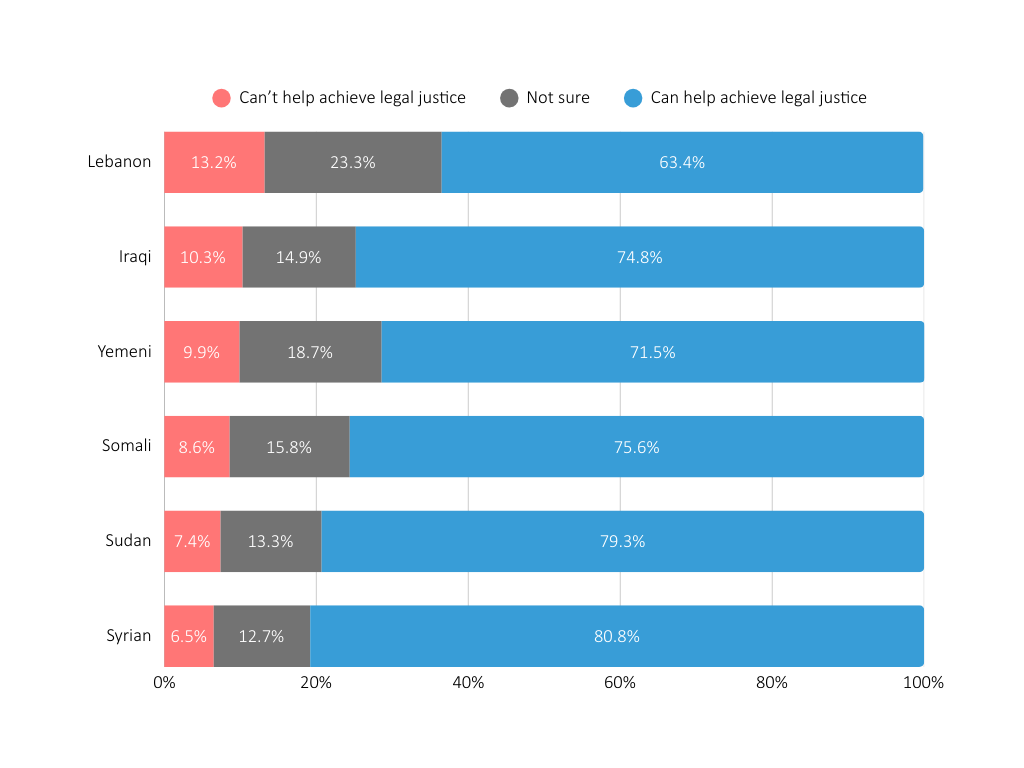
**

**Figure 2. Perception that collecting mortality data in conflict-affected areas can help achieve legal justice and hold perpetrators accountable by country (p < .001)**

**Table 1. Variables associated with willingness to participate in mortality studies in conflict-affected areas**

|  | | **Would you be willing to participate in a survey about war-related mortality in your country? n (%)** | | **P value** (Pearson Chi-Square/ Mann-Whitney U) |
| --- | --- | --- | --- | --- |
|  |  | **Yes** | **No/Not sure** |  |
| Age | Median (IQR) | 30 (24–38) | 27 (22–35) | <0.001 |
| Sex | Female | 1267 (64.9%) | 686 (35.1%) | 0.008 |
|  | Male | 1039 (69.2%) | 463 (30.8%) |  |
| Nationality | Sudanese | 523 (77.5%) | 152 (22.5%) | <0.001 |
|  | Iraqi | 488 (73.6%) | 175 (26.4%) |  |
|  | Syrian | 299 (71.7%) | 118 (28.3%) |  |
|  | Yemeni | 467 (69.7%) | 203 (30.3%) |  |
|  | Somali | 193 (55.3%) | 156 (44.7%) |  |
|  | Lebanese | 336 (49.3%) | 345 (50.7%) |  |
| Education | I have never attended school | 12 (63.2%) | 7 (36.8%) | 0.473 |
|  | Primary | 30 (58.8%) | 21 (41.2%) |  |
|  | Secondary | 244 (63.5%) | 140 (36.5%) |  |
|  | Diploma | 168 (65.4%) | 89 (34.6%) |  |
|  | Bachelor's | 1358 (67.2%) | 663 (32.8%) |  |
|  | Master | 353 (67.2%) | 172 (32.8%) |  |
|  | Doctorate or higher | 141 (71.2%) | 57 (28.8%) |  |
| Present in your country during the period of war or armed conflict | Yes | 1945 (67.8%) | 923 (32.2%) | 0.003 |
|  | No | 361 (61.5%) | 226 (38.5%) |  |
| How comfortable are you with sharing mortality data during wars or armed conflicts on social media platforms | Comfortable | 1073 (77.7%) | 308 (22.3%) | <0.001 |
|  | Neutral | 708 (61.0%) | 453 (39.0%) |  |
|  | Uncomfortable | 525 (57.5%) | 388 (42.5%) |  |
| Do you think collecting this type of data could be harmful in any way? | Yes | 311 (68.7%) | 142 (31.3%) | <0.001 |
|  | Not sure | 625 (60.0%) | 417 (40.0%) |  |
|  | No | 1370 (69.9%) | 590 (30.1%) |  |
| Do you think there are cultural or religious beliefs that make it difficult to collect mortality data in your country | Yes | 469 (71.3%) | 189 (28.7%) | <0.001 |
|  | Not sure | 705 (59.0%) | 489 (41.0%) |  |
|  | No | 1132 (70.6%) | 471 (29.4%) |  |

**Table 2. Variables associated with perceived importance of local leaders’ permission before collecting mortality data in conflict-affected areas**

|  | | **Do you think it is important to obtain permission from local community leaders before collecting mortality data in their community? n (%)** | | **P value** (Pearson Chi-Square/ Mann-Whitney U) |
| --- | --- | --- | --- | --- |
|  |  | **Yes** | **No/Not sure** |  |
| Age | Median (IQR) | 29 (24–37) | 29 (23–37) | 0.934 |
| Sex | Female | 811 (41.5%) | 1142 (58.5%) | 0.015 |
|  | Male | 686 (45.7%) | 816 (54.3%) |  |
| Nationality | Somali | 197 (56.4%) | 152 (43.6%) | <0.001 |
|  | Yemeni | 370 (55.2%) | 300 (44.8%) |  |
|  | Sudanese | 309 (45.8%) | 366 (54.2%) |  |
|  | Syrian | 172 (41.2%) | 245 (58.8%) |  |
|  | Lebanese | 256 (37.6%) | 425 (62.4%) |  |
|  | Iraqi | 193 (29.1%) | 470 (70.9%) |  |
| Education | I have never attended school | 10 (52.6%) | 9 (47.4%) | 0.478 |
|  | Primary | 24 (47.1%) | 27 (52.9%) |  |
|  | Secondary | 152 (39.6%) | 232 (60.4%) |  |
|  | Diploma | 120 (46.7%) | 137 (53.3%) |  |
|  | Bachelor's | 867 (42.9%) | 1154 (57.1%) |  |
|  | Master | 239 (45.5%) | 286 (54.5%) |  |
|  | Doctorate or higher | 85 (42.9%) | 113 (57.1%) |  |
| Present in your country during the period of war or armed conflict | Yes | 1264 (44.1%) | 1604 (55.9%) | 0.051 |
|  | No | 233 (39.7%) | 354 (60.3%) |  |
| Do you think there are cultural or religious beliefs that make it difficult to collect mortality data in your country | Yes | 344 (52.3%) | 314 (47.7%) | <0.001 |
|  | Not sure | 479 (40.1%) | 715 (59.9%) |  |
|  | No | 674 (42.0%) | 929 (58.0%) |  |

**Table 3. Factors associated with trust in reporting mortality data to governmental institutions during conflict**

|  | | **Participant trust in government institutions for sharing mortality data during conflict n (%)** | | **P value** (Pearson Chi-Square/ Mann-Whitney U) |
| --- | --- | --- | --- | --- |
|  |  | **Trust it** | **Don’t trust it** |  |
| Age | Median (IQR) | 29 (23-35) | 30 (24-38) | <0.001 |
| Sex | Female | 819 (41.9%) | 1134 (58.1%) | 0.002 |
|  | Male | 710 (47.3%) | 792 (52.7%) |  |
| Nationality | Lebanese | 341 (50.1%) | 340 (49.9%) | <0.001 |
|  | Somali | 170 (48.7%) | 179 (51.3%) |  |
|  | Syrian | 201 (48.2%) | 216 (51.8%) |  |
|  | Iraqi | 303 (45.7%) | 360 (54.3%) |  |
|  | Sudanese | 278 (41.2%) | 397 (58.8%) |  |
|  | Yemeni | 236 (35.2%) | 434 (64.8%) |  |
| Education | I have never attended school | 10 (52.6%) | 9 (47.4%) | 0.051 |
|  | Primary | 16 (31.4%) | 35 (68.6%) |  |
|  | Secondary | 177 (46.1%) | 207 (53.9%) |  |
|  | Diploma | 98 (38.1%) | 159 (61.9%) |  |
|  | Bachelor's | 886 (43.8%) | 1135 (56.2%) |  |
|  | Master | 255 (48.6%) | 270 (51.4%) |  |
|  | Doctorate or higher | 87 (43.9%) | 111 (56.1%) |  |


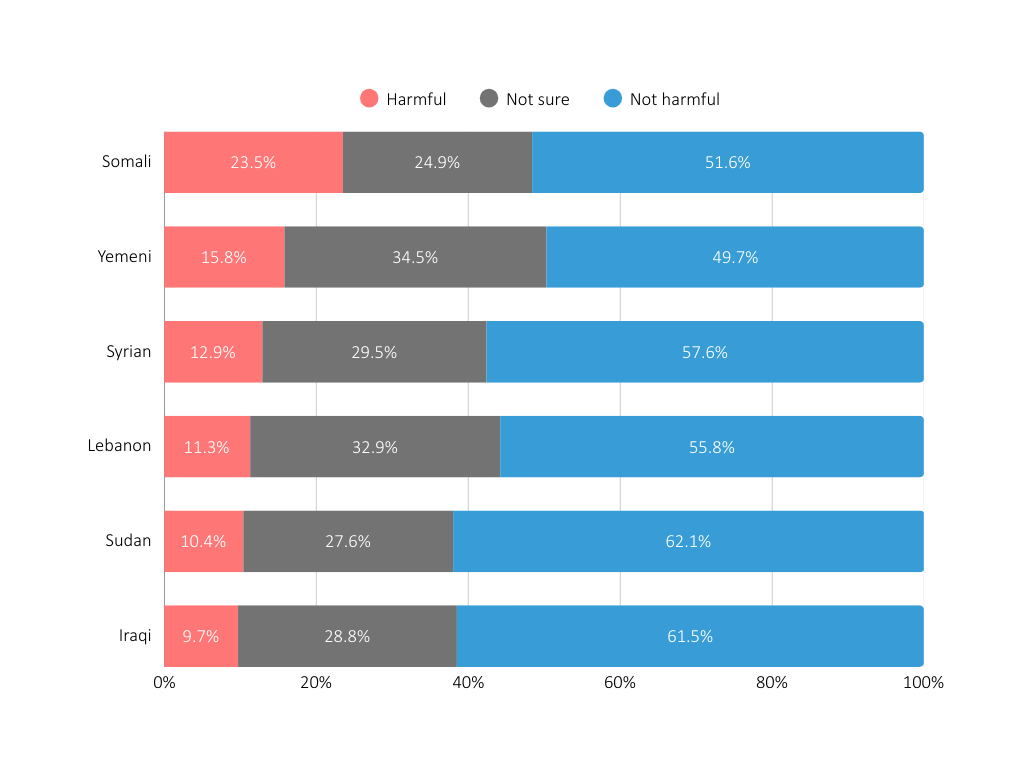
**Figure 3. Perceived harm of collecting mortality data in conflict-affected areas by country (p < .001)**


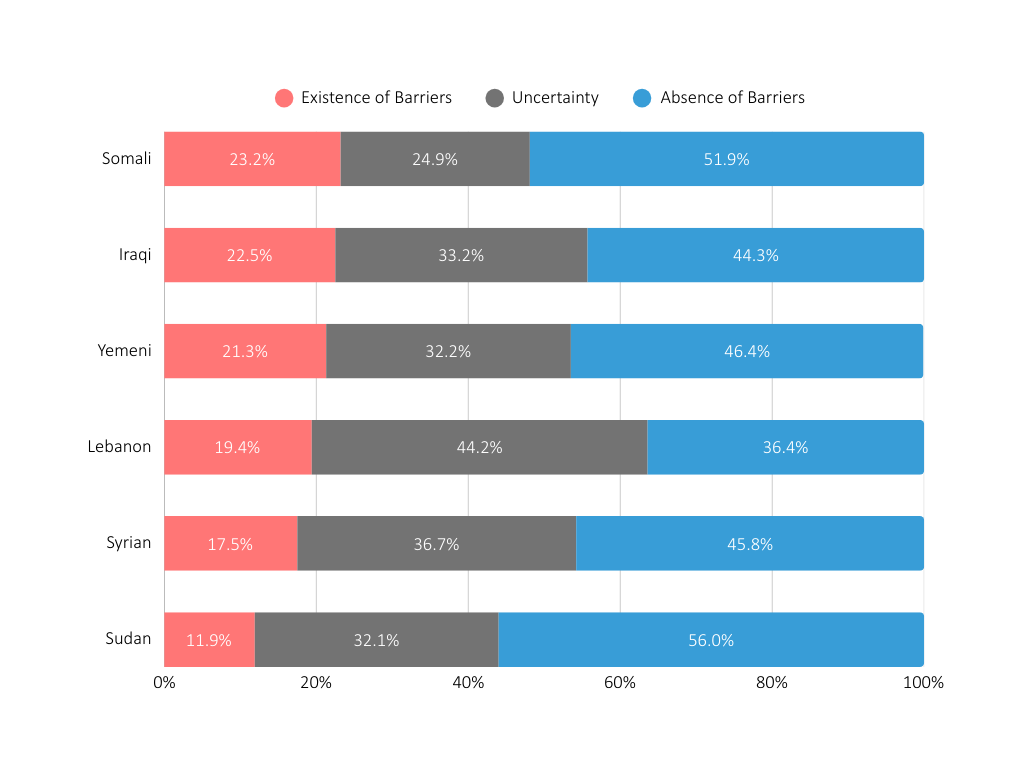
**Figure 4. Perception of cultural or religious beliefs that hinder mortality data collection by country (p < .001)**

**Table 4. Participants’ responses to open-ended questions**

|  | **n (%)** |
| --- | --- |
| **Why do you think collecting mortality data during wars and armed conflicts is important (N=2776)** |  |
| Monitor and document the effects of conflict, including human and asset losses | 1658 (59.7) |
| Support affected families by providing compensation and preserving the right to know the fate of missing people | 317 (11.4) |
| Ensure legal accountability and strengthen human rights | 255 (9.2) |
| Improve humanitarian and public health response, and prevent deaths | 172 (6.2) |
| Increase public awareness and enhance media engagement | 130 (4.7) |
| Support peacebuilding and facilitate political mobilization | 108 (3.9) |
| Guide resource allocation and inform governmental decisions during the war and post-war period | 90 (3.2) |
| Support research | 46 (1.7) |
| **Why do you trust the entity you selected? (N=** **2143)** |  |
| Impartiality, non-partisanship, credibility, trustworthiness, transparency, and have good reputation | 846 (39.5) |
| Possesses governmental, legal, or official authority and represent the people | 412 (19.2) |
| Have the power to change and disseminate the information | 237 (11.1) |
| Ensures data security and integrity | 172 (8.0) |
| Have the professional capacity and the experience required | 155 (7.2) |
| Locally grounded and understands the context of the communities | 132 (6.2) |
| Has a humanitarian and peace-driven mission | 102 (4.8) |
| Internationally recognized | 87 (4.1) |
| **Why do you not trust the other organizations? (N=** **1844)** |  |
| Lack of Credibility, Ethical misconduct, Political Bias, or Hidden Agendas | 1130 (61.3) |
| Safety and security concerns | 159 (8.6) |
| Lack of competence or capacity | 129 (7.0) |
| Preference for government or official bodies | 125 (6.8) |
| Unknown to me | 103 (5.6) |
| Previous experience | 96 (5.2) |
| Distance from ground reality | 58 (3.1) |
| Others | 44 (2.4) |
| **How can collecting information about death in conflict can be harmful? (N=** **244)** |  |
| Retaliation | 121 (49.6) |
| Political manipulation and national security threats | 78 (32.0) |
| Social and psychological impact and fear | 45 (18.4) |
| **What are the cultural or religious beliefs that make it difficult to collect mortality data in your country (N=** **198)** |  |
| Challenges that stem from ethnic, tribal, sectarian, and religious diversity | 121 (61.1) |
| Cultural secrecy, fear of defamation, and honor preservation ^1^ | 30 (15.2) |
| Privacy of death and sanctity of the deceased ^2^ | 19 (9.6) |
| Sensitivity regarding female deaths, including sexual assaults and honor crimes. | 13 (6.6) |
| Belief in divine justice (for martyrs) and acceptance of fate | 8 (4.0) |
| Others | 7 (3.5) |

^1^ This category includes fears of social stigma, shame, and defamation associated with naming the deceased or revealing the causes of death

^2^ This include discussing the cause of death or conducting post-mortem procedures is often seen as a violation of religious norms and an intrusion on the personal and familial dignity of the deceased.

**Table 5. Participants’ responses to open-ended questions by country**

| **Why do you think collecting mortality data during wars and armed conflicts is important n (%)** | | | | | | |
| --- | --- | --- | --- | --- | --- | --- |
|  | **Sudan** | **Yemen** | **Syria** | **Lebanon** | **Iraq** | **Somalia** |
| Monitor and document the effects of conflict, including human and asset losses | 351 (58.2) | 369 (60.3) | 188 (54.3) | 286 (67.0) | 327 (59.5) | 137 (57.6) |
| Support affected families by providing compensation and preserving the right to know the fate of missing people | 61 (10.1) | 69 (11.3) | 54 (15.6) | 51 (11.9) | 65 (11.8) | 17 (7.1) |
| Ensure legal accountability and strengthen human rights | 61 (10.1) | 62 (10.1) | 49 (14.2) | 26 (6.1) | 40 (7.3) | 17 (7.1) |
| Improve humanitarian and public health response, and prevent deaths | 50 (8.3) | 43 (7.0) | 14 (4.1) | 11 (2.6) | 35 (6.4) | 19 (8.0) |
| Increase public awareness and enhance media engagement | 31 (5.1) | 20 (3.3) | 24 (6.9) | 25 (5.9) | 24 (4.4) | 6 (2.5) |
| Support peacebuilding and facilitate political mobilization | 20 (3.3) | 23 (3.8) | 11 (3.2) | 10 (2.3) | 28 (5.1) | 16 (6.7) |
| Guide resource allocation and inform governmental decisions during the war and post-war period | 20 (3.3) | 19 (3.1) | 5 (1.5) | 13 (3.0) | 20 (3.6) | 13 (5.5) |
| Support research | 9 (1.5) | 7 (1.1) | 1 (0.3) | 5 (1.2) | 11 (2.0) | 13 (5.5) |
| **Why do you trust the organization you selected? n (%)** | | | | | | |
|  | **Sudan** | **Yemen** | **Syria** | **Lebanon** | **Iraq** | **Somalia** |
| Impartiality, non-partisanship, credibility, trustworthiness, transparency, and have good reputation | 189 (41.5) | 182 (39.1) | 110 (41.0) | 130 (42.1) | 190 (41.4) | 45 (24.2) |
| Possesses governmental, legal, or official authority and represent the people | 64 (14.1) | 83 (17.8) | 44 (16.4) | 59 (19.1) | 122 (26.6) | 40 (21.5) |
| Have the power to change and disseminate the information | 65 (14.3) | 51 (10.9) | 37 (13.8) | 21 (6.8) | 33 (7.2) | 30 (16.1) |
| Ensures data security and integrity | 27 (5.9) | 47 (10.1) | 15 (5.6) | 24 (7.8) | 36 (7.8) | 23 (12.4) |
| Have the professional capacity and the experience required | 25 (5.5) | 32 (6.9) | 21 (7.8) | 31 (10.0) | 29 (6.3) | 17 (9.1) |
| Locally grounded and understands the context of the communities | 36 (7.9) | 36 (7.7) | 11 (4.1) | 20 (6.5) | 15 (3.3) | 14 (7.5) |
| Has a humanitarian and peace driven mission | 37 (8.1) | 20 (4.3) | 12 (4.5) | 11 (3.6) | 17 (3.7) | 5 (2.7) |
| Internationally recognized | 12 (2.6) | 15 (3.2) | 18 (6.7) | 13 (4.2) | 17 (3.7) | 12 (6.5) |
| **Do you not trust the other organizations? n (%)** | | | | | | |
|  | **Sudan** | **Yemen** | **Syria** | **Lebanon** | **Iraq** | **Somalia** |
| Lack of Credibility, Ethical misconduct, Political Bias, or Hidden Agendas | 237 (66.0) | 275 (62.3) | 113 (51.1) | 177 (65.6) | 254 (64.5) | 74 (45.7) |
| No theme | 31 (8.6) | 34 (7.8) | 31 (14.0) | 17 (6.3) | 17 (4.3) | 29 (17.9) |
| Safety and security concerns | 23 (6.4) | 37 (8.5) | 19 (8.6) | 13 (4.8) | 22 (5.6) | 15 (9.3) |
| Lack of competence or capacity | 16 (4.5) | 30 (6.9) | 16 (7.2) | 20 (7.4) | 27 (6.8) | 16 (9.9) |
| Preference for government or official bodies | 13 (3.6) | 21 (4.8) | 11 (5.0) | 16 (5.9) | 32 (8.1) | 10 (6.2) |
| Unknown to me | 20 (5,6) | 18 (4.1) | 16 (7.2) | 5 (1.9) | 32 (8.1) | 5 (3.1) |
| Previous experience | 9 (2.5) | 9 (2.1) | 10 (4.5) | 16 (5.9) | 8 (2.0) | 6 (3.7) |
| Distance from ground reality | 10 (2.8) | 13 (3.0) | 5 (2.3) | 6 (2,2) | 3 (0.8) | 7 (4.3) |
| **How can collecting information about death in conflict can be harmful? n (%)** | | | | | | |
|  | **Sudan** | **Yemen** | **Syria** | **Lebanon** | **Iraq** | **Somalia** |
| Retaliation | 24 (60) | 31 (56.4) | 17 (54.8) | 15 (42.9) | 17 (44.7) | 17 (37.8) |
| Political manipulation and national security threats | 9 (22.5) | 18 (32.7) | 12 (38.7) | 10 (28.6) | 13 (34.2) | 16 (35.6) |
| Social and psychological impact and fear | 7 (17.5) | 6 (10.9) | 2 (6.5) | 10 (28.6) | 8 (21.1) | 12 (26.7) |
| **What are the cultural or religious beliefs that make it difficult to collect mortality data in your country n (%)** | | | | | | |
|  | **Sudan** | **Yemen** | **Syria** | **Lebanon** | **Iraq** | **Somalia** |
| Challenges that stem from ethnic, tribal, sectarian, and religious diversity | 6 (25) | 23 (63.9) | 22 (75.9) | 17 (58.6) | 46 (76.7) | 7 (35.0) |
| Cultural secrecy, fear of defamation, and honor preservation: This category includes fears of social stigma, shame, and defamation associated with naming the deceased or revealing the causes of death | 9 (37.5) | 2 (5.6) | 3 (10.3) | 5 (17.2) | 3 (5.0) | 8 (40.0) |
| Privacy of death and sanctity of the deceased: This include discussing the cause of death or conducting post-mortem procedures is often seen as a violation of religious norms and an intrusion on the personal and familial dignity of the deceased. | 3 (12.5) | 4 (11.1) | 3 (10.3) | 3 (10.3) | 4 (6.7) | 2 (10.0) |
| Sensitivity regarding female deaths, including sexual assaults and honor crimes | 5 (20.8) | 3 (8.3) | 1 (3.5) | 1 (3.5) | 3 (5.0) | 0 (0) |
| Belief in divine justice (for martyrs) and acceptance of fate | 1 (4.2) | 2 (5.6) | 0 (0) | 0 (0) | 1 (1.7) | 3 (15.0) |
| Others | 0 (0) | 2 (5.6) | 0 (0) | 3 (10.3) | 3 (5.0) | 0 (0) |
